# Deciphering the impacts of gut and tissue microbiome on human cancers

**DOI:** 10.1002/cnr2.1924

**Published:** 2023-11-14

**Authors:** Mukul S. Godbole, Vishal Singh

**Affiliations:** ^1^ Department of Biosciences and Technology, Faculty of Sciences and Health Sciences Dr. Vishwanath Karad MIT World Peace University Pune India; ^2^ Department of Nutritional Sciences The Pennsylvania State University University Park Pennsylvania USA

Cancer, a multifactorial culmination, is one of the leading causes of death in men and women globally. Exposure to one or more cancer‐causing agents, such as smoking, alcohol, alkylating agents, radiations, and secondary risk factors, such as obesity or certain infections, work together to promote cancer development. Our current mechanistic understanding of cancer development suggests that environmental factors, including atypical microbiota, influence cancer susceptibility, particularly in individuals who are genetically predisposed to cancer development. Approximately 15%–20% of human cancers are causally linked with microbial pathogens. Recent findings from taxonomic and metabolomics analyses of gut microbiota and microbial communities residing within tumors have enhanced our understanding of microbiome‐dependent regulation of carcinogenesis. The potential of gut microbiota to modulate the efficacy and toxicity of anticancer drugs further expands the significance of gut microbiota in cancer prevention and therapy, warranting in‐depth discussion. The “Cancer and Microbiome” special issue encompasses original research and review articles that provide insights into the recent understanding of the role of the gut microbiota in cancer, including the promises of microbiome‐based therapies for cancer treatment. This special issue also emphasizes the role of microbial products that influence host metabolism and the circulation of host metabolites that may influence cancer incidence and progression.

The gut microbiota is a complex ecosystem of trillions of bacteria, archaea, viruses, and fungi that reside in the digestive tract. These microbes have been known to help the host metabolize complex food materials and pharmaceutical agents, including anticancer drugs. Moreover, a balanced and functional gut microbial community trains and fine‐tunes immunological and cellular responses to kill invading pathogens and initiate a cancer‐fighting immune response. Therefore, a disruption of this intricate relationship has been implicated in a variety of neoplasms. Unsurprisingly, cancers arising in the gastrointestinal tract have also been thought to have a close association with microbes residing in the gut. Beneficial modulation of gut microbiota through the intake of dietary fibers—nondigestible complex carbohydrates—has gained enormous attention. A research group led by Dr. Bhisham Narayan Singh and Dr. Ashwini Kumar reviewed the benefits of prebiotics—fermentable dietary fibers that stimulate the growth of beneficial bacteria in the gut—for overall health and cancers of the gastrointestinal tract.^1^ The authors elaborated on the interactions between gut microbiota and prebiotics, and how these interactions influence cancer growth and improve the functions of immune system. They also described the effects of individual and combined prebiotics on gastrointestinal tract cancers, wherein individual components including inulin have been described to have overall beneficial effects on human health. In contrast, a research group led by Dr. Vishal Singh observed that refined dietary fiber inulin, commonly present in processed foods, causes abnormal accumulation of succinate in the gut, which leads to colon inflammation and tumorigenesis in a mouse model.^2^ They have previously shown that inclusion of inulin‐enriched high‐fat diet promotes hepatocellular carcinoma in mice. Here, the authors discuss that refined inulin may selectively enrich particular bacterial groups that enhance colonic inflammation. Thus, these studies highlight the necessity to thoroughly evaluate the influence of refined dietary fibers that are claimed to have health‐promoting effects. Further, a group led by Dr. Indranil Chattopadhyay reviewed the landscape of gut microbes associated with gastric cancer.^3^ They have elaborated on the roles of virulence factors secreted by several bacteria, such as *Escherichia coli, Lactobacillus*, *Streptococcus*, and so on, in addition to *Helicobacter pylori*, which lead to inflammation and development of gastric cancer. Moreover, they have explained the possibility of using microbial metabolites as future biomarkers for diagnosis and prognosis of gastric cancer.

Breast cancer is the most heterogeneous and amongst the most frequently diagnosed cancers in women worldwide. A change in lifestyle, along with genetic and physiological predisposition, has increased the propensity of incidence of breast cancer and mortality with the disease. Interestingly, despite being distant from and seemingly unrelated to the breast, alterations in the microbial composition of the gastrointestinal tract have profound effects on the development and functions of the breasts in women, including development and progression of cancer. A review article by a group led by Dr. Sneha Joshi and Dr. Rupa Mishra explains the differences in the gut and breast tissue microbial composition on the occurrence of breast cancer subtypes.^4^ They have emphasized on the effects of microbial composition on immune responses. Moreover, the authors have highlighted the role of artificial intelligence and machine learning on the diagnosis and prognosis of breast cancer using microbial dysbiosis as a biomarker. Further, a group led by Dr. Mukul S. Godbole has reviewed the importance of gut microbiome on the levels of circulating estrogen and progesterone that influence the incidence and progression of breast cancer.^5^ While previous studies by Dr. Godbole have emphasized on the anticancer effects of progesterone, the present review provides a thorough investigation of how progesterone may confer this effect in humans via metabolic activities of the gut microbiome. Their review also describes the interindividual differences in response to anticancer therapies conferred by the gut microbiome, highlighting the necessity of a diverse microbiome for complete response to chemo‐, immune‐, and hormonal‐therapy.

Efficient immune responses form the basis for counteracting pathogenic invasions. The host microbiota, especially of the gut, helps maintain an overall intact immunity. However, chronic alterations in the microbial diversity immensely impact host immune responses, and thus the occurrence of diseased conditions. A review article by a group led by Dr. Asmita Das explains this phenomenon in gastrointestinal cancers with a special emphasis on immune‐related receptors, such as toll‐like receptors and NOD‐like receptors.^6^ An alteration in the functions of the immune‐related receptors has been correlated with cancer incidence. Thus, the authors propose that it is imperative to ensure a healthy, intact tissue microbiota to help prevent pathogenic infections.

Tobacco and human papillomavirus (HPV) infection were considered among the primary causes of cancers arising in the head and neck region. However, recent investigations have elucidated a strong association between oral microbiome and head and neck cancers. While interindividual differences in microbial compositions are minimal, a dysbiosis of the oral microbiome can alter the metabolism of key carcinogenic substances and also cause chronic infections in the head and neck regions that lead to the formation of cancers. A review articulated by Dr. Sanket Desai explains the landscape of genomic and epigenomic alterations induced by oral microbiome, comprising bacteria, fungi, and various viruses, such as HPV, Epstein–Barr virus, and hepatitis B and C viruses.^7^ These changes include somatic copy number changes, expression changes, genomic deletions, and global hypomethylation. Previously, Dr. Desai has described the role of *Fusobacterium* in the progression of HPV‐negative oral cancer. While head and neck cancers have largely been grouped into HPV‐positive and HPV‐negative groups, efforts like these should enable future studies to rectify this binary groupism into a more elaborate distribution of cancers.

All articles published in this special issue highlight the limitations of our current understanding of the diverse effects of gut and tissue microbes on the occurrence of various cancers. Articles also express the need to develop strategies that can counterbalance the dysbiotic conditions that lead to chronic inflammation of host cells, and thus cancer incidence or progression. While the causal association with cancer remains to be established for a majority of the microorganisms, the close interactions warrant more focused research on elucidating the influence of microbes on cancer. We sincerely hope that this special issue will help to advance our understanding of the microbiome's involvement in cancer development and therapeutics, as well as increase awareness among clinicians, researchers, and the general public. A thorough understanding of this diverse and intricate interaction may help to improve the treatment of cancers and clinical outcomes in the future.

## AUTHOR CONTRIBUTIONS

Mukul S. Godbole: Writing–original draft, and writing–review and editing; Vishal Singh: Writing–original draft, and writing–review and editing.

## CONFLICT OF INTEREST STATEMENT

The authors have stated explicitly that there are no conflicts of interest in connection with this article.

## ETHICS STATEMENT

Not applicable.

## Data Availability

Data sharing is not applicable to this article as no datasets were generated or analyzed during the current study.
